# Drivers of immersive virtual reality adoption intention: a multi-group analysis in chemical industry settings

**DOI:** 10.1007/s10055-021-00586-3

**Published:** 2021-10-07

**Authors:** Ryo Toyoda, Fernando Russo Abegão, Sue Gill, Jarka Glassey

**Affiliations:** 1 School of Engineering, Newcastle University, Newcastle upon Tyne, UK; 2 Learning and Teaching Development Service, Newcastle University, Newcastle upon Tyne, UK

**Keywords:** Virtual reality adoption, UTAUT 2 multi-group analysis, Chemical industry, Training, Adoption intention, PLS-SEM

## Abstract

The present study uses the modified Unified Theory of Acceptance and Use of Technology 2 to examine the effect of factors such as performance expectancy (PE), effort expectancy (EE), social influence (SI), and hedonic motivation (HM) that may motivate operators and employees to adopt IVR-based technology into their training. Results of a multi-group analysis based on nationality, prior IVR experience, and/or length of work experience, to analyse the potential similarities and/or differences in perception and acceptance towards using IVR-based technology are also presented. The quantitative research data were gathered using an online questionnaire from 438 chemical operators and/or employees who either speak German, French, or English. Partial least squares structural equation modelling and multi-group analysis based on SmartPLS™ version 3 were used to carry out the path and multi-group analyses. The results show that the behavioural intention (BI) towards adoption of IVR was influenced by PE, EE, and HM for all abovementioned subpopulation. However, the relationship of SI to BI was not supported for respondents with prior IVR experience and for respondents coming from Western region. Although Henseler’s-based multi-group PLS analysis reveals that there was no significant difference between the group comparisons, it is still important to take into account these socio-demographic factors as there are definite group differences in terms of the ranking order of each construct for the IVR adoption intentions among each subpopulation. The implications and future directions were discussed.

**Supplementary Information:**

The online version contains supplementary material available at 10.1007/s10055-021-00586-3.

## Introduction

Since several companies around the world are adapting and embracing the concept of industry 4.0, technologies such as virtual reality (VR) technology gained a significant level of attention and created a paradigm shift in several areas of training in the fields of chemical (Colombo et al. 2014), medical (Bissonnette et al. 2019), and aviation industries (Clifford et al. 2019). As pointed out by many researchers, training materials such as PowerPoint presentations or pre-recorded lectures only provide and explain instructions and rules without realistic feeling for the given scenarios (Arkorful and Abaidoo 2015). Such approaches are not particularly effective, especially in the abovementioned fields (Dholakiya et al. 2019). Creating realistic training scenarios frequently require users to be trained in situations that involve risk-taking methodologies. For instance, it is impossible to mimic or even simulate an explosion in a chemical plant due to cost, safety, and environment implications (Manca et al. 2013). As VR technology, particularly immersive virtual technology (IVR), can provide users with a safe 3D training environment space, promoting knowledge acquisition through active involvement, it is possible to create a representation of real-life scenario for training under normal or abnormal situations within a safe setting while retaining stress drivers (Bissonnette et al. 2019; Dholakiya et al. 2019).

The use of IVR technology in chemical industry setting can improve higher-order thinking competencies that are important for scenario-based training, such as problem-solving and communication skills. Nevertheless, it is still necessary to investigate the perceptions and acceptance of users towards the application of IVR technologies in chemical industries. This is due to the fact that this specific area scarcely uses the applied theories on technology acceptance, which provide answers whether it is appropriate to use IVR or not. Since the success rate of employing IVR technology is dependent on the number of people who are eager to try and use this technology, it is vital to identify the factors affecting IVR adoption intentions (Van Slyke et al. 2007). For this purpose, the Unified Theory of Acceptance and Use of Technology 2 (UTAUT 2) model, an extension of the UTAUT model, will be used in this study. Both the UTAUT and UTAUT 2 models proved to be more comprehensive and give higher explanatory power than other models, as validated by Venkatesh et al. (2003, 2012), respectively. However, the main reason for choosing the UTAUT 2 model is that it allows understanding the adoption and usage intention of the consumers (i.e. employees and operators) towards IVR technology in the chemical industries. The UTAUT 2 model considers seven key factors: performance expectancy (PE), effort expectancy (EE), social influence (SI), facilitating conditions (FC), hedonic motivation (HM), price value (PV), and habit (H). The model also includes three moderators: age, gender, and experience. Both the key factors and the moderators are considered to affect the behavioural intention (BI) and/or use behaviour (USE). In this model, all the seven key factors affect the behavioural intention, while the key factors FC, H, and BI influence the use of behaviour.

Previous studies investigated the acceptance of IVR in various fields. For instance, Hartl and Berger (2017) explored the consumer acceptance of VR glasses in entertainment content (e.g. watching 360° documentary video and playing 3-min game) using the extended UTAUT 2 model. They found out that only 3 out of 6 factors (i.e. PE, SI, and H) showed significant effects on behavioural intention to adopt IVR system (Hartl and Berger 2017). On the other hand, Kunz and Santomier (2019) used the extended UTAUT 2 model to investigate the acceptance of VR technology in sport content reported that only 3 out of 7 factors (i.e. PE, SI, and HM) showed significant effects on behavioural intention to adopt IVR system (Kunz and Santomier 2019). As observed, the IVR adoption intention results from both studies are different. The results obtained from these IVR adoption studies cannot be used to generalise other groups as it may cause misinterpretation due to the differences in terms of sample demographics and fields of application of the technology. Hence, this study employs a modified UTAUT 2 model to investigate the factors for the IVR adoption from the perspective of operators and employees in the chemical industry.

Sarstedt et al. (2011) proposed that data assessment should incorporate socio-demographic data (e.g. age, gender, nationality, experience) into the partial least square (PLS) model as interpreting results from the single population (i.e. homogeneous representation of all observations), eliminating the population heterogeneity, gives misleading outcomes. Hence, this study uses partial least square multi-group analysis (PLS-MGA) to test the similarities and differences between the different sub-categories of chemical operators and employees in terms of their intentions to adopt IVR. The findings from this model will prove useful for understanding the acceptance of IVR among operators and employees after considering their background. Through this, chemical industries will be able to implement more effective training programmes in futures and more judiciously consider investments in IVR technology for health and safety training.

## State of the art and hypothesis development

### Virtual reality in chemical industry

Since chemical processes are usually highly complex in nature, operators, and employees are required to undergo a series of effective training sessions to ensure the safe operation for each piece of equipment in a chemical plant (Nazir et al. 2012). In the past, a combination of various training methods such as classroom training, on-job training, and/or simulation tools was considered an effective training method to enhance the required skills for operators and employees (Patle et al. 2014). However, for highly automated chemical plants, these methods alone are not sufficient for training professionals (Colombo et al. 2014).

For the operators and employees to enhance their capability and potential to deal both common and critical situations, it is important to consider training methods that enable staff to cooperate as they would do in real situations. The methods should also allow staff to experience and understand normal and dangerous plant conditions in order to enhance their anticipation of circumstances (Colombo et al. 2011; Manca et al. 2013). Moreover, as it can be dangerous, impractical, and too costly to recreate these situations in an actual plant, the new training methods must provide real feel scenarios during the training while keeping people safe (Nazir et al. 2012).

More immersive, safe, and innovative tools, such as virtual reality (VR), technologies are increasingly used in the highly complex and automated chemical industries especially in safety education and training (Colombo et al. 2014; Manca et al. 2013; Nazir et al. 2012). The term “virtual reality (VR) refers to an interactive and immersive computer-based three-dimensional (3D) synthetic environment that can simulate real experiences in which the user can interact with various components through multiple sensorial channels” (Burdea 2003). VR can be categorised as non-immersive, semi-immersive, or fully immersive, depending on the quality of the immersion experience (Cronin 1997). Of these three categories, fully immersive virtual reality (IVR) is the best option in terms of the sense of presence since it allows the users to be totally secluded from the real world and thus providing a better understanding of certain skills or knowledge (Fällman et al. 1999).

Over the years, some researchers have made significant advances in using VR technology for training in the chemical industry. For instance, Colombo et al. (2014) created a training scenario on how to respond to pressurised liquid butane (C4) leakage due to inadvertent excavator operation in IVR. Their study showed that operators e trained using VR performed 50% better in fault diagnosis than those trained with conventional slide-supported presentations (Colombo et al. 2014). Moreover, Garcia Fracaro et al. (2021) are recreating the step-by-step procedure on how to use various equipment and how to respond to incidents in n-butyllithium (n-BuLi) production process.

### Theoretical underpinning and hypothesis development

Although the awareness and popularity of using new technologies like e-learning and VR have increased especially during the COVID-19 period, the adoption of these new technologies is still relatively low. It is thus important to investigate the interrelationship between influential factors and behavioural intention. Without understanding the gap between what people claim through their attitudes and involvement and how they behave, the rate of succession in implementing IVR training will be low. Given the relevance and growing importance of IVR adoption intention in chemical industry, it is timely to examine the perceptions of operators and employees towards the intentions of IVR adoption for H&S training.

This study has employed the UTAUT 2, an adaptation of UTAUT to a consumer context, to explain and predict behavioural intention of users towards IVR adoption. As mentioned earlier, the UTAUT model is deemed the well-known model in information technology (IT) user acceptance research since it has been synthesised from the empirical comparison of eight models and validated using within-subject longitudinal data from different organisations (Li and Kishore 2006; Venkatesh et al. 2003).

Several studies confirmed that the abovementioned key factors have a significant influence on BI to adopt new technology, such as an e-scooter VR service (Huang 2020), and head-mounted VR displays in learning (Shen et al. 2019). As this study focused purely on perception of IVR adoption from the perspective of operators and employees in the chemical industry, the construct of PE has been conceptualised as the extent to which chemical operators and employees perceive IVR as a tool that would lead to additional improvement in their job performance. The construct of EE has been conceptualised as the extent to which chemical operators and employees perceive IVR to be simple to operate and easy to use. The construct of SI has been conceptualised as the extent to which chemical operators and employees perceive the expectations of their peers on their use of IVR. Finally, the construct of HM has been conceptualised as the extent to which chemical operators and employees perceive IVR as a tool to bring additional joy and enjoyment. From these, the following hypotheses, adapted and modified from Venkatesh et al. (2003, 2012), are proposed:

- *H1* PE will have a significant influence on BI to adopt IVR for H&S learning.
- *H2* EE will have a significant influence on BI to adopt IVR for H&S learning.
- *H3* SI will have a significant influence on BI to adopt IVR for H&S learning.
- *H4* HM will have a significant influence on BI to adopt IVR for H&S learning.

Authors such as Hartl and Berger (2017) and Shen et al. (2019) used the UTAUT model to identify the effect of different constructs towards IVR adoption intention using a single population sample. However, according to Sarstedt et al. (2011), interpretation of results from a homogenous population could be misleading as every individual has their own perceptions and evaluations of outcomes. Thus, they proposed to assess data by adding more subgroups of data into the model to minimise misinterpretation of results (Sarstedt et al. 2011).

Various authors considered socio-demographic variables such as age, gender, nationality, and experience in an analysis of the behaviour of technology users. For instance, Venkatesh and his co-workers clustered participants based on their age, gender, and experience and analysed the willingness of users to accept and use new technology in the workplace, and the willingness of consumers to accept and use mobile internet technology using UTAUT and UTAUT 2 model, respectively. Both of these studies confirmed that socio-demographic variables were key factors in the BI to adopt and/or use new technology. On the other hand, Palau-Saumell et al. (2019) employed the extended UTAUT 2 model to compare the usage intention with mobile application for restaurant searches and/or reservations, and Ramirez-Correa et al. (2015) used the Technology Acceptance Model (TAM) to compare e-learning intentions. These authors confirmed that some of the socio-demographic variables (i.e. gender and age) were not key factors in the BI of users to adopt and/or use these technologies. Since there is some discrepancy among previous studies on the effect of socio-demographic variables based on the technology used, it is important to establish the effect of these socio-demographic variables in the behaviour of users towards IVR adoption. By doing so for different groups, the influence of the PE, EE, SI, and HM in terms of IVR adoption intention can be compared. Thus, the following hypotheses were proposed:

- *H5* The influence of adoption in IVR will be different between groups based on the nationality of the employees.
- *H6* The influence of adoption in IVR will be different between groups based on the prior IVR experience of the employees.
- *H7* The influence of adoption in IVR will be different between groups based on the length of work experience of the employees.

**Fig. 1:**
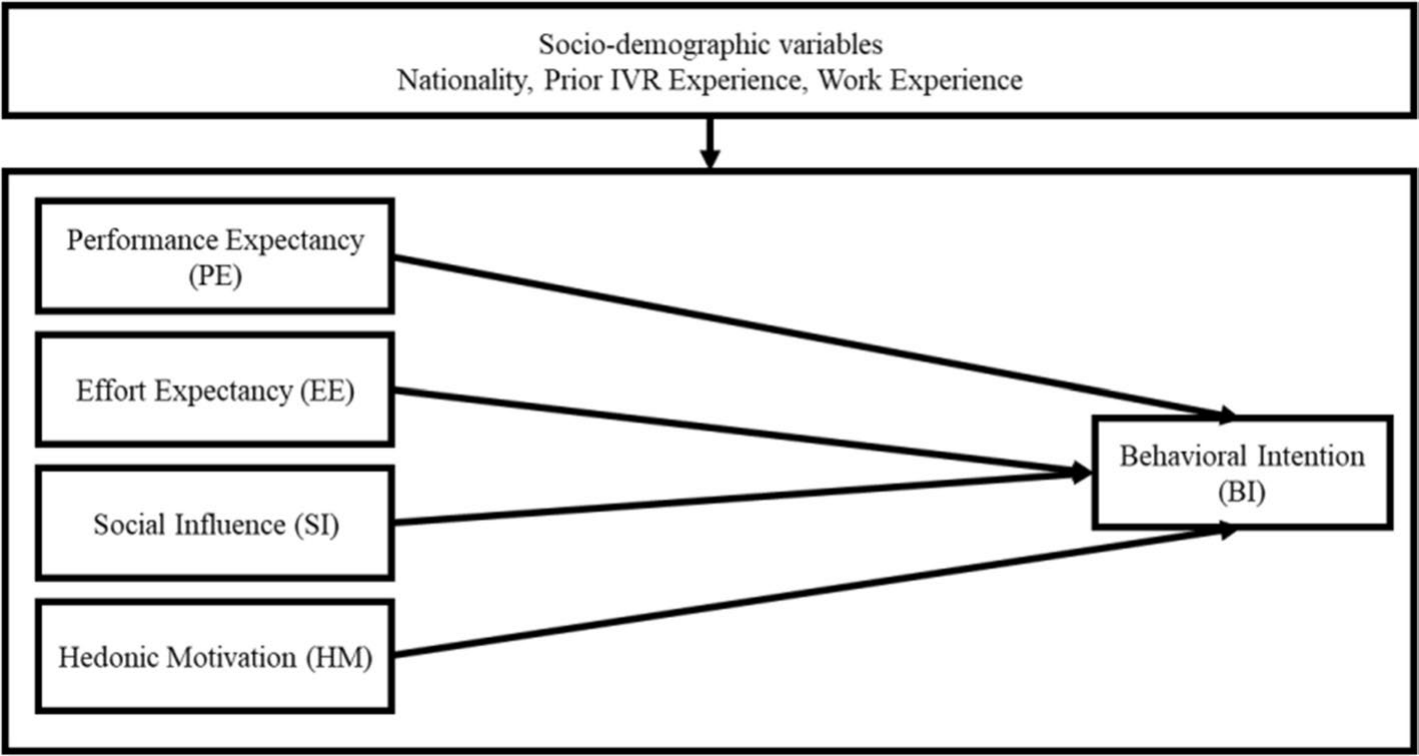
The modified UTAUT 2 model

Finally, a conceptual model on IVR adoption intention modified from UTAUT 2 model is postulated as shown in Fig. 1.

## Research methodology

### Questionnaire design

To measure the perceptions of chemical industry employees towards H&S training using IVR technology, this study employed an online questionnaire survey. The final online questionnaire comprised of two sections. The first section covered the socio-demographic background of the respondents, including their gender, age, nationality, prior IVR experience, and length of employment. The second section contained the items about PE, EE, SI, HM, and BI that were adopted from previously reported research using the UTAUT 2 model and that were verified as valid and reliable (Venkatesh et al. 2012). For each item, some of the words were modified to better fit the scope of IVR games in training. The respondents indicated their agreement with each item on a six-point Likert scale, ranging from 1 for strongly disagree to 6 for strongly agree. The reason for choosing the 6-point Likert scale is that it gives a higher trend of discrimination and reliability as compared to a 5-point Likert scale (Chomeya 2010).

Since this study also aimed to examine the difference in IVR adoption between Eastern and Western countries, questionnaires in English, French, and German were prepared. The questionnaire was originally created in English and was subsequently translated into French and German by a native speaker. A separate native speaker then performed a blind back-translation of the questionnaire into English, which was compared with the initial English version to ensure the uniformity and validity of the translation (Dorer 2012). The English list of items used in the study with its corresponding constructs is shown in Table 1.

**Table 1:** Lists of measurement items used in the study

Latent variable	Item	Explanation
Performance expectancy (PE)	PE_1	I think that using the VR environment will be useful for practicing H&S procedures
	PE_2	Using VR environment will probably enable me to learn the H&S procedures more quickly
	PE_3	If I use this VR environment, I will improve my performance on H&S procedures
Effort expectancy (EE)	EE_1	I think using the VR environment will be clear and understandable
	EE_2	I think that it will be easy for me to operate the platform in which the VR environment is running
Social influence (SI)	SI_1	I think that the organisation will support me in learning how to use the VR environment
	SI_2	People who influence my behaviour at work think that I should use this VR environment
	SI_3	I think my supervisor will be very supportive of the use of this VR environment for my job
Hedonic motivation (HM)	HM_1	I feel that it will be a bad idea to use the VR environment for H&S training
	HM_2	I think that the actual process of using the VR environment for H&S training is fun
	HM_3	I think that using VR environment for H&S training will be very frustrating
Behavioural intention (BI)	BI_1	If made available to me, I would recommend using the VR environment for learning to apply the H&S procedures to my colleagues
	BI_2	If made available to me, I plan to continue to use VR environment for H&S training frequently
	BI_3	I think that after using the VR for H&S training, I will be ready to use this learning environment for another training course

### Data collection process

Before the data collection, the questionnaire was tested by academic experts in the field (cf. acknowledgements section) to ensure face validity as well as content validity. The modified questionnaire was then pilot-tested for readability using postgraduate chemical engineering students and volunteer engineers in the chemical industry. Prior to the distribution of the questionnaire, an ethical approval was obtained from the Ethics Committee at the university.

The responses were collected from employees working in chemical industries situated in Europe and Asia. Since it was impossible to include all employees working in the country in the sample, convenience sampling, a non-probability sampling method was used. Four hundred thirty-eight (438) completed questionnaires were collected and were subjected to data screening to eliminate invalid questionnaires. Since no incomplete or duplicated data were present, all responses were used for data analysis.

The power analysis using G*Power 3 analysis software was performed to ensure the sufficiency of the sample size (Faul et al. 2007). From the calculation, the minimum required sample size for this study is 129. Thus, the sample of 438 collected data used in this study is sufficient.

### Data analysis

Structural Equation Modelling (SEM) was used in this study since it enables simultaneous analysis of the hypothesised relationships in a given model, and also possible correlations between multiple dependent and independent variable (Hair et al. 2017). Since this study intended to explore the modified version of the well-known technology acceptance theory (UTAUT2 model), it is more appropriate to use partial least squares structural equation modelling (PLS-SEM). PLS-SEM has the advantage of relying on a lower sample size and non-normally distributed data requirements compared to covariance-based (CB) SEM (Hair et al. 2017). Therefore, PLS-SEM using SmartPLS™ version 3 software was used to assess the measurement model and to test the path relationship between the constructs of the model based on the collected data (Ringle et al. 2015).

Given that the aim of this study is to examine the differences in hypothetical relationships between groups, a multi-group analysis approach (MGA) in PLS-SEM was carried out. The overall sample was divided into groups based on the categorical variable of interest. The analysis of the measurement invariance (i.e. equivalence) of composite models (MICOM) across two or more groups was then carried out following a three-step procedure: (1) configural invariance, (2) compositional invariance, and (3) the equality of composite mean values and variances (Henseler et al. 2016). After establishing measurement invariance, comparison of path coefficients among groups using Henseler PLS-MGA procedure was evaluated to determine the significant differences between groups (Matthews 2017).

## Results

### Participant profile

The demographic information of the participants is summarised in Table 2. Out of the 438 participants, those coming from Eastern countries (i.e. Asia) account for 32.9% of the group compared to the participants coming from Western countries (i.e. Europe) representing 67.1% of the group. Males account for 66.4% of the participants, while females account for 33.6%. The majority of the participants are between the ages of 20–39 (57.3%) and have more than five (5) years of working experience (62.8%). Finally, more than 70% of the participants had prior experience in playing video games, but only 35.4% of them have tried head-mounted display VR.

**Table 2:** Demographic information of participants (*n* = 438)

Characteristics	Items	Frequency	Percentage
Nationality	Eastern countries	144	32.9
	Western countries	294	67.1
Gender	Male	291	66.4
	Female	147	33.6
Age	20–29	155	35.4
	30–39	96	21.9
	40–49	83	18.9
	50–59	88	20.1
	60 and above	16	3.7
Working experience	Less than a year	37	8.4
	1–5 years	126	28.8
	6–20 years	153	34.9
	More than 20 years	122	27.9
Experience to VR	Yes	155	35.4
	No	283	64.6
Experience to video game	Yes	326	74.4
	No	112	25.6

### Assessment of the measurement model

In the measurement model using a PLS analysis, the validation guidelines of Hair et al. (2017) were used to examine the reliability and validity of the constructs along with their corresponding items.

To test the internal consistency reliability, Cronbach’s alpha and composite reliability (CR) was calculated. The calculated values of internal consistency and convergent validity for participants based on nationality, prior IVR experience, and length of work experience are shown in Table A.1 (supplementary information document). These values for all factors were above the minimum cut-off 0.6 (Hair et al. 2017), which indicates that the constructs have strong internal consistency reliability for each considered subpopulation.

In order to test the construct validity, the convergent and discriminant validities were calculated. According to Hair et al. (2017), the calculated value for the average variance extracted (AVE) and the factor loading should be greater than 0.5 and 0.708, respectively. Table A.1 shows that all constructs in every subpopulation considered had an average variance extracted (AVE) and factor loading values higher than the minimum cut-off. Furthermore, the Heterotrait-Monotrait ratio (HTMT) was calculated to evaluate discriminant validity. The calculated values of discriminant validity for participants based on nationality, prior IVR experience, and length of work experience are shown in Table A.2 (supplementary information document). The calculated confidence interval of the HTMT statistics was lower than the threshold value of 0.9 for all combination of constructs (Gold et al. 2001). Thus, the results obtained indicate adequate convergent and discriminant validities for each considered subpopulation.

### Assessment of measurement invariance

Before performing the multi-group analysis (PLS-MGA) to determine the potential similarities and/or differences between path coefficients of the subpopulation considered, measurement invariance should be tested (Henseler et al. 2016). This is required to ensure that a given measure is interpreted in a conceptually similar matter across a specified population (Horn and Mcardle 1992). In PLS-SEM, measurement invariance can be tested using measurement invariance of composites (MICOM) procedure which includes configural invariance, compositional invariance, and equality of composite mean values and variances (Henseler et al. 2016). According to Henseler et al. (2016), completing these three steps will give a full measurement invariance (i.e. pooling data of different groups), but establishing the first two steps is sufficient to conduct PLS-MGA.

The assessment of configural invariance involves evaluation of the measurement models for all groups to check if the same number of indicators and the same variance-based model estimation were used and if all the indicators were treated equally across the specified groups (Henseler et al. 2016). As the analysis and assessment of the measurement models (reliability and validity) for all groups was completed in the previous sub-section, configural invariance was established.

To ensure the homogeneity of the composite scores across the considered subpopulations, compositional invariance was examined using a permutation analysis with 5000 resamples through SmartPLS 3 software (Henseler et al. 2016; Ringle et al. 2015). The calculated values of MICOM for participants based on nationality, prior IVR experience, and length of work experience are shown in Table A.3 (supplementary information document). All of the values of *c* (compositional invariance correlation) were close to 1 and fell within the 95% confidence interval. Hence, the compositional invariance was established across all the subpopulation groups. Subsequently, upon the establishment of both configural and compositional invariance, partial measurement invariance, which is the minimum required to conduct the PLS-MGA was achieved.

**Table 3:** Results of full collinearity test for each subpopulation

Construct	Variance inflation factors (VIF)					
	Western	Eastern	With prior IVR experience	Without prior IVR experience	< 5 year work experience	> 5 year work experience
PE	2.283	1.785	1.632	2.634	2.043	2.237
EE	2.016	1.973	1.678	2.257	2.219	1.976
SI	1.298	1.299	1.143	1.360	1.297	1.290
HM	2.063	1.488	1.433	2.068	1.748	1.918

*PE* performance expectancy, *EE* effort expectancy, *SI* social influence, *HM* hedonic motivation, *BI* behavioural intention

### Assessment of the structural model and PLS-MGA results

The structural model was evaluated for every subpopulation based on the collinearity assessment, the coefficient of determination (*R*^*2*^), and the path coefficient significance (*β*). Before calculating *R*^*2*^ and *β*, it is important to check first if there are no issues connected with multi-collinearity (Hair et al. 2017). To do this, full collinearity variance inflation factors (VIFs) were evaluated. Table 3 shows that since the obtained VIF values for PE, EE, SI, and HM were significantly below the threshold value of 3, multi-collinearity issues were not a concern.

**Fig. 2:**
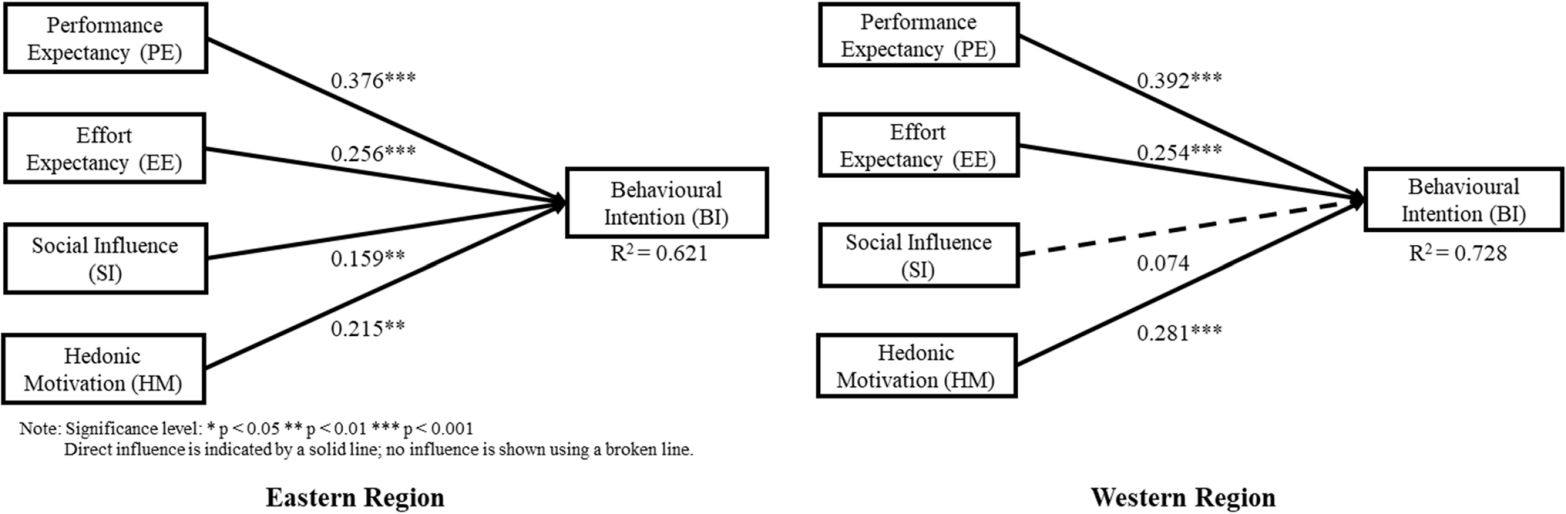
Structural equation model of the employees’ perception on IVR games in training based on nationality

**Fig. 3:**
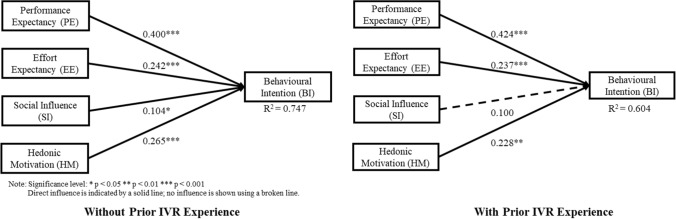
Structural equation model of the employees’ perception on IVR games in training based on prior IVR experience

**Fig. 4:**
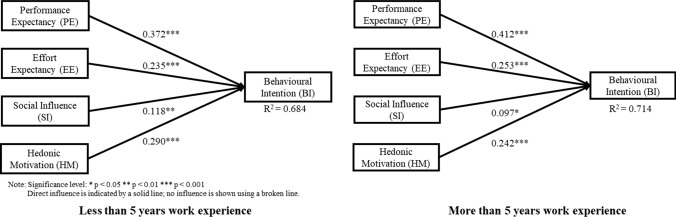
Structural equation model of the employees’ perception on IVR games in training based on length of work experience

The structural model that specifies the correlations between the constructs for each subpopulation was evaluated by investigating the path significance using a bias-correlated and accelerated (BCa) bootstrapping without sign change re-sampling technique based on 5000 sub-sample (Hair et al. 2017; Ringle et al. 2015). BCa bootstrapping was used to handle the issue of peaked and skewed distribution by adjusting the confidence intervals for skewness (Efron 1987). The path coefficients and the extent of influence on the structural equation model for every subpopulation are shown in Figs. 2, 3, and 4.

Figures 2, 3, and 4 show the variance explained by the PE, EE, SI, and HM constructs for behavioural intention to adopt IVR was 0.621, 0.728, 0.747, 0.604, 0.684, and 0.714 for the group from the Eastern region, Western region, without prior IVR experience, with prior IVR experience, less than 5 years of work experience, and more than 5 years of work experience, respectively. Since *R*^*2*^ value > 0.2 is considered acceptable in the behavioural study, all models possess adequate capacity to explain BI to adopt IVR (Hair et al. 2017).

Table 4 summarises the outcomes of the path coefficients values for each subpopulation. H1, which suggested significant relationships between PE and BI, was validated in all groups, and the values were above 0.37. H2, which suggested significant relationships between EE and BI, was validated in all groups, and the values were above 0.23. H3, which suggested significant relationships between SI and BI, was not validated in the western group nor in the prior IVR experience group, but was validated in the other groups. H4, which suggested significant relationships between HM and BI, was validated in all groups, and the values were above 0.21.

**Table 4:** Outcomes of the structural equation model multi-group analysis

Relationship	Based on nationality			
	Eastern	Western	│Diff│	Henseler’s MGA *p* value
H1: PE → BI	0.376***	0.392***	0.016	0.566
H2: EE → BI	0.256***	0.254***	0.002	0.482
H3: SI → BI	0.159**	0.074	0.084	0.108
H4: HM → BI	0.215**	0.281***	0.066	0.795

Relationship	Based on prior experience to IVR			
	Without IVR Experience	With IVR Experience	│Diff│	Henseler’s MGA *p* value
H1: PE → BI	0.400***	0.424***	0.024	0.605
H2: EE → BI	0.242***	0.237***	0.005	0.477
H3: SI → BI	0.104*	0.100	0.004	0.474
H4: HM → BI	0.265***	0.228**	0.037	0.319

Relationship	Based on length of work experience			
	< 5 years	> 5 years	│Diff│	Henseler’s MGA *p* value
H1: PE → BI	0.372***	0.412***	0.041	0.685
H2: EE → BI	0.235***	0.253***	0.018	0.587
H3: SI → BI	0.118**	0.097*	0.021	0.370
H4: HM → BI	0.290***	0.242***	0.049	0.251

*Diff.* path coefficient differences, *PE* performance expectancy, *EE* effort expectancy, *SI* social influence, *HM* hedonic motivation, *BI* behavioural intention

Significance level of path coefficient: *p < 0.05; **p < 0.01; ***p < 0.001

Having evaluated the measurement and structural model, Henseler’s MGA (PLS-MGA), a non-parametric test, was used to assess the similarities and differences of path coefficients between the groups. In this method, if the MGA *p* value is less than 0.05 or greater than 0.95, there is a 5% level significant difference between specific path coefficients between two subpopulations. The outcome of the PLS-MGA *p* values in Table 4 shows that there were no significant group differences between any of the subpopulation groups (e.g. based on nationality, prior IVR experience, and length of work experience). Therefore, H5, H6, and H7 were not accepted.

## Discussion

The current study applied a group-based approach to examine the perception of chemical operators and employees towards IVR adoption intentions. This was done by using a modified version of UTAUT 2 that is composed of PE, EE, SI, and HM constructs. Upon comparison of the groups of chemical operators and employees, based on nationality, prior IVR experience, and work experience using PLS-MGA, there were several similarities and differences in the relationships investigated in the current study.

### Theoretical implications

This study provides meaningful insights for the current literature on IVR adoption based on the UTAUT 2 model. The explanatory power, determined by the *R*^*2*^ value for each of the group-specific structural equation models, has values greater than 60.4%, indicating that the proposed research model is able to predict well the IVR adoption intentions of chemical operators and employees. Thus, future practitioners such as instructors and researchers can use this methodology based on the modified UTAUT 2 model on IVR adoption intention to test its reliability and validity in other settings.

Concerning hypothesis testing, the empirical results for all subpopulations (Western, Eastern, with and without prior IVR experience, less than and more than 5 years of work experience) showed that performance expectancy significantly influences the IVR adoption intention in chemical industries. Moreover, among the four key factors, PE was the strongest factor in the respondents' influencing BI to adopt IVR for all subpopulations (Figs. 2, 3, 4). This result is also consistent with previous studies that confirmed the significant influence of PE on sport VR context acceptance (Kunz and Santomier 2019) and on E-mail acceptance (Mao and Palvia 2008). Hsu and Lin (2008) reported that PE plays critical factor in work-related environment and given that the respondents considered in this study were chemical operators and employees, this suggests that regardless of the group, respondents perceive that using IVR technology in chemical industry training would increase their job performance.

In addition, effort expectancy was found to significantly influence the IVR adoption intention for all subpopulations. The results show that regardless of the group, respondents are driven to adopt IVR if they perceive the IVR experience as easy and simple. This outcome is also consistent with the findings on mobile internet acceptance (Venkatesh et al. 2012) and online virtual tour-guiding platform acceptance (Chiao et al. 2018). However, the rank order for EE is different per subpopulation. For instance, subpopulations, such as Eastern group, with prior IVR experience group, and more than 5 years of work experience group, show that EE is the second strongest factor influencing BI to adopt IVR. According to Ramayah et al. (2005), EE is considered to have more influence in BI for less experienced users but in this study, this is not true. This is may be because VR is still not as accessible as other technologies such as desktop computers or smartphones. It is also possible that the respondents with prior IVR experience may still have expected that it would not be easy, and it would take quite some time for them to master the controls in the IVR environment. Moreover, compared to less than 5 years of work experience or western group, respondents who have more than 5 years of experience or come from the Eastern region are more hesitant to learn the IVR training. Elgohary and Abdelazyz (2020) reported that there is a significant difference between the length of work experience and the resistance to change in a developing country setting. Given these observations, it is understandable that for these groups, EE is a more important factor to consider for IVR adoption intention than SI or HM.

Hedonic motivation is significantly influenced by the IVR adoption intention for all subpopulations. The results show that, regardless of the group, respondents are driven to adopt IVR if they perceive the IVR experience as fun and entertaining. This outcome is also consistent with previous studies on the IVR adoption intentions for an e-scooter service (Huang 2020), and the acceptance of social telepresence robots (Han and Conti 2020). However, the rank order for HM is different per subpopulation. For instance, subpopulations such as Western group, without prior IVR experience group, and less than 5 years of work experience group, all show that HM is the second strongest factor influencing BI to adopt IVR. As mentioned by Venkatesh et al. (2012), as experience intensifies, the attractiveness of the novelty and innovativeness will lessen which in return, affect the HM. Given this argument, it is possible that respondents who do not have prior IVR experience are more eager to experience the IVR technology than the respondents with prior IVR experience. Thus, what makes it more appealing and fun for the former group is because of its innovative features and modernity. This is also true for respondents with less than 5 years of work experience as well as for the Western group. This may be because respondents from these groups are more willing to go out of their comfort zones and are more open when trying new and innovative things such as IVR technology. Given these reasons, for the abovementioned groups, even though IVR technology will be mainly used for learning procedural know-how, HM is a more essential factor than SI or EE, when considering IVR adoption intention.

Although the rank order was lowest for social influence in subpopulations such as Eastern, without prior IVR experience, regardless of the length of work experience, it significantly impacts IVR adoption intention in chemical industries for these groups. This outcome is also consistent with previous studies that confirmed the significant influence of SI on e-governance of users (Alraja 2016), and also on health IT (Bozan et al. 2016) adoption intentions. Similar to the “bandwagon effect”, people tend to adopt new technology if it works favourably for their respected peers and/or supervisors (Tsai et al. 2013). This effect is especially true in a situation where implementation of new technology is still in its initial stage as reported by Alraja, (2016). Thus, this suggests that for these groups, peer influence is still considered to be an important factor in determining what people should take on since implementation of IVR is in its initial stage. However, subpopulations such as Western, and with prior IVR experience groups do not support this hypothesis. Researchers such as Teo and Noyes (2014), also reported that SI was found to be not significant on BI to use technology among younger pre-service teachers as they were digital natives and tended to choose for themselves whether to use the given technology or not. For the two groups mentioned above, it is possible that subjects within these groups were already aware of the existence of IVR technology (i.e. digital natives). This suggests that they do not need to be influenced by their peers or supervisors as they know the capabilities of IVR technology. Thus, it is important to consider the subpopulation as the construct of SI changes over time.

The modified UTAUT2 is helpful to explore more factors that influence the intentions of chemical operators and employees to adopt IVR in a different setting. Through using this model, the relationships between BI and PE, EE, SI, and HM were verified. The results of the analysis and comparison of multi-group analysis using Henseler’s MGA analysis revealed that there was no significant difference on the models between the effect of PE, EE, SI, and HM on BI to adopt IVR in groups of chemical operators and employees based on nationality, prior IVR experience, or length of work experience. Nevertheless, it is still necessary to take into account these socio-demographic factors as there are definite group differences in terms of the ranking order of each construct for the IVR adoption intentions among each subpopulation. Incorporating PLS-SEM and MGA methods is beneficial since these methods are not limited to analysing IVR adoption behaviour of the population sample but also useful in determining group differences (Matthews 2017; Ramírez-Correa et al. 2015).

### Practical implications

In terms of the practical implications, this study will be able to inform the chemical industry policymakers/decision-makers in a number of ways. If the institutions decide to create an IVR-based training, they can consider the ranking order of each construct to design appropriate training strategies in the IVR environment to satisfy the needs of the users. Through this, managerial and training staff, and corporate policymakers will have a clearer view on what should be implemented, allowing them to decide whether to emphasise game elements, more easy controls, more procedural aspects, etc. Purchase the IVR system from other industries can also be guided through the use of the results of this model, as a basis to choose the most suitable IVR system and to make a fair judgement regarding the IVR system specifications that lead to a more effective delivery of the training programme. As the COVID-19 pandemic continues to develop through multiple waves around the world, online courses, as well as training, are becoming the new normal. It is thus expected, that IVR will play a significant role in delivering professional development and health and safety training. Thus, the current study recommends that it is important to consider constructs such as PE, EE, SI, and HM as key factors in determining the adoption rate of IVR technology.

### Limitations and future research

Although this study provides deeper understanding to researchers and stakeholders, it is important to acknowledge some limitations. First, responses were obtained from convenience sampling as most of the chemical industries do not openly publish the details of their employees to ensure compliance with privacy regulations. For the future studies, it would be beneficial to replicate this study using different groups from a wider and a more heterogeneous population (e.g. chemical industries from different parts of the world and different sectors) to establish the robustness of the results. Second, as this study only used a quantitative statistical approach to examine the relationship between factors, the exploration of qualitative approaches or combination of both methods may be beneficial to further investigate the mechanism of behavioural intention among this group of people. Finally, since the perception of users with respect to IVR adoption in chemical industry may change over time, longitudinal studies at a various timeline of IVR acceptance process to reinvestigate the IVR adoption considering other key constructs may be valuable.

## Conclusions

This study aimed to compare the factors influencing the adoption intention of IVR using the modified version of UTAUT 2 model. The IVR adoption intention of predefined groups of chemical operators and employees were analysed using PLS-SEM and multi-group analysis (MGA) with SmartPLS 3.0. Although the results of PLS-MGA did not show statistically significant differences between the predefined groups of respondents, the MGA approach is effective in understanding the intentions of multiple groups. This study presented recommendations for the chemical industry policymakers in formulating suitable strategies on possible ways to implement IVR-based technology from the measured groups.

## Supplementary Information

Below is the link to the electronic supplementary material.

Supplementary file1 (PDF 140 kb)
