# Supplementary material for: Drivers of immersive virtual reality adoption intention: a multi-group analysis in chemical industry settings
Source: Virtual Real. 2021 Oct 7:1–12. Online ahead of print. doi: 10.1007/s10055-021-00586-3 (PMC8494627; doi:10.1007/s10055-021-00586-3)
Supplement: Supplementary file 1 — Supplementary file1 (PDF 140 kb) [file 10055_2021_586_MOESM1_ESM.pdf]

Appendix A – Supplementary document

Table A.1. Internal consistency reliability and convergent validity analysis of participants.

Constructs	Items	Factor Loading			Cronbach's Alpha			Composite Reliability			Average Variance Extracted		
		Nat[a]	Prior IVR EXP[b]	Work EXP[c]	Nat[a]	Prior IVR EXP[b]	Work EXP[c]	Nat[a]	Prior IVR EXP[b]	Work EXP[c]	Nat[a]	Prior IVR EXP[b]	Work EXP[c]
Performance Expectancy	PE_1	0.907 (0.915)	0.893 (0.917)	0.913 (0.909)	0.893 (0.903)	0.880 (0.910)	0.913 (0.892)	0.934 (0.939)	0.926 (0.943)	0.945 (0.933)	0.824 (0.838)	0.806 (0.847)	0.851 (0.822)
	PE_2	0.918 (0.915)	0.909 (0.926)	0.933 (0.912)									
	PE_3	0.899 (0.917)	0.892 (0.919)	0.922 (0.899)									
Effort Expectancy	EE_1	0.892 (0.898)	0.880 (0.906)	0.886 (0.898)	0.725 (0.711)	0.625 (0.757)	0.712 (0.723)	0.879 (0.873)	0.841 (0.892)	0.874 (0.878)	0.784 (0.775)	0.726 (0.804)	0.776 (0.782)
	EE_2	0.879 (0.862)	0.823 (0.887)	0.876 (0.871)									
	EE_3*												
Social Influence	SI_1	0.765 (0.822)	0.723 (0.774)	0.767 (0.773)	0.680 (0.796)	0.634 (0.743)	0.710 (0.712)	0.824 (0.880)	0.803 (0.853)	0.837 (0.839)	0.610 (0.710)	0.577 (0.660)	0.632 (0.635)
	SI_2	0.753 (0.856)	0.711 (0.825)	0.807 (0.775)									
	SI_3	0.823 (0.850)	0.839 (0.836)	0.811 (0.840)									
Hedonic Motivation	HM_1	0.866 (0.798)	0.830 (0.860)	0.807 (0.865)	0.809 (0.682)	0.672 (0.804)	0.718 (0.795)	0.886 (0.815)	0.816 (0.882)	0.833 (0.879)	0.722 (0.595)	0.597 (0.714)	0.624 (0.708)
	HM_2	0.824 (0.750)	0.722 (0.825)	0.795 (0.803)									
	HM_3	0.859 (0.766)	0.763 (0.849)	0.767 (0.854)									
Behavioural Intention	BI_1	0.923 (0.935)	0.918 (0.928)	0.917 (0.928)	0.904 (0.894)	0.878 (0.912)	0.876 (0.912)	0.940 (0.934)	0.925 (0.945)	0.924 (0.945)	0.839 (0.826)	0.804 (0.851)	0.802 (0.851)
	BI_2	0.934 (0.945)	0.904 (0.953)	0.916 (0.946)									
	BI_3	0.890 (0.842)	0.866 (0.886)	0.852 (0.893)									

Note: * - Removed due to the lack of outer loading reliability (< 0.7) [a] - based on the nationality [b] - based on the prior IVR experience [c] - based on the length of work experience

Numbers in bracket at [a]- Values for Eastern countries group Numbers in bracket at [b]- Values for without prior experience to IVR group Numbers in bracket at [c]- Values for more than 5 year work experience group

Table A.2. Discriminant validity analysis using Heterotrait-Monotrait (HTMT) ratio of participants.

	**PE**			**EE**			**SI**			**HM**			**BI**
	**Nat[a]**	**Prior IVR EXP[b]**	**Work EXP[c]**	**Nat[a]**	**Prior IVR EXP[b]**	**Work EXP[c]**	**Nat[a]**	**Prior IVR EXP[b]**	**Work EXP[c]**	**Nat[a]**	**Prior IVR EXP[b]**	**Work EXP[c]**	
PE													
EE	0.813 (0.767)	0.746 (0.847)	0.819 (0.808)										
SI	0.568 (0.470)	0.452 (0.561)	0.538 (0.528)	0.595 (0.555)	0.426 (0.641)	0.552 (0.597)							
HM	0.781 (0.571)	0.553 (0.794)	0.636 (0.766)	0.804 (0.691)	0.756 (0.767)	0.799 (0.764)	0.523 (0.148)	0.306 (0.473)	0.231 (0.491)				
BI	0.867 (0.771)	0.785 (0.882)	0.824 (0.861)	0.887 (0.832)	0.830 (0.889)	0.895 (0.871)	0.590 (0.518)	0.466 (0.608)	0.542 (0.581)	0.844 (0.646)	0.687 (0.832)	0.779 (0.805)	

Note: The numbers indicates the pairwise correlations between variables PE = Performance Expectancy; EE = Effort Expectancy; SI = Social Influence; HM = Hedonic Motivation; BI = Behavioural Intention
[a] - based on the nationality [b] - based on the prior IVR experience [c] - based on the length of work experience
Numbers in bracket at [a]- Values for Eastern countries group Numbers in bracket at [b]- Values for without prior experience to IVR group Numbers in bracket at [c]- Values for more than 5 year work experience group

Table A.3. Assessment of measurement invariance of composite models (MICOM) test of participants.

**Construct**	Based on nationality			Based on prior IVR experience			Based on length of work experience		
	**Step 1**								
	Configural Invariance								
**PE**	**Yes**			**Yes**			**Yes**		
**EE**	**Yes**			**Yes**			**Yes**		
**SI**	**Yes**			**Yes**			**Yes**		
**HM**	**Yes**			**Yes**			**Yes**		
**BI**	**Yes**			**Yes**			**Yes**		
	**Step 2**								
**Construct**	Correlation c	95% confidence interval	Compositional invariance	Correlation c	95% confidence interval	Compositional invariance	Correlation c	95% confidence interval	Compositional invariance
**PE**	1.000	[0.999, 1.000]	**Yes**	1.000	[0.999, 1.000]	**Yes**	1.000	[0.999, 1.000]	**Yes**
**EE**	1.000	[0.997, 1.000]	**Yes**	1.000	[0.997, 1.000]	**Yes**	1.000	[0.997, 1.000]	**Yes**
**SI**	0.998	[0.984, 1.000]	**Yes**	0.997	[0.983, 1.000]	**Yes**	0.998	[0.985, 1.000]	**Yes**
**HM**	0.997	[0.992, 1.000]	**Yes**	0.999	[0.992, 1.000]	**Yes**	0.996	[0.993, 1.000]	**Yes**
**BI**	1.000	[0.999, 1.000]	**Yes**	1.000	[0.999, 1.000]	**Yes**	1.000	[0.999, 1.000]	**Yes**

Note: PE = Performance Expectancy; EE = Effort Expectancy; SI = Social Influence; HM = Hedonic Motivation; BI = Behavioural Intention
